# Factors contributing to compliance with Expanded Programme on Immunization and RTS, S/AS01 schedules among children aged 24–40 months in Central Tongu District of Ghana

**DOI:** 10.1186/s12936-025-05469-5

**Published:** 2025-10-23

**Authors:** Diana Sena Awutey-Hinidza, Margaret Kweku, Thomas Gyan, Martin Adjuik, John Paul Amenu, Jonathan Mawutor Gmanyami, Kwaku P. Asante

**Affiliations:** 1 Central Tongu District Health Directorate, Ghana Health Service, Central Tongu, Ghana; 2 Fred N. Binka School of Public Health, Epidemiology and Biostatistics Department, University of Health and Allied Sciences, Hohoe, Ghana; 3 Kintampo Health Research Centre (KHRC), Research and Development Division, Ghana Health Service, Kintampo, Ghana; 4 Global Health and Infectious Diseases Research Group, Kumasi Centre for Collaborative Research in Tropical Medicine, Kumasi, Ghana; 5 School of Public Health, Kwame Nkrumah University of Science and Technology, Kumasi, Ghana

**Keywords:** Caregiver, Children, Malaria, *RTS,S/AS01*, Vaccine, Immunization, Compliance, Schedules, Expanded Program Immunization, Ghana, Volunteer

## Abstract

**Background:**

The Expanded Programme on Immunization (EPI) is the most successful global childhood programme in reducing mortality and morbidity from vaccine-preventable diseases. Introducing the *RTS, S/AS01* malaria vaccine into the routine health system added additional vaccination visits at 24 months. This study determined the factors contributing to compliance with EPI, including *RTS, S/AS01* malaria vaccine schedules in the Central Tongu District of Ghana.

**Methods:**

A matched case–control study (1:1) was undertaken between December 2021 and February 2022 among caregivers of children aged 24 to 40 months. Mantel-Haenzel odds ratio and conditional logistic regression analysis were used to determine the strength of association between the level of compliance and independent variables.

**Results:**

Of the 220 caregivers,110 were cases, and 110 were controls. Good knowledge about vaccines and vaccination schedules among caregivers was 62% (n = 139). Caregivers who earned income 46% (OR_MH_ 0.54, 95% CI 0.29–1.00, p = 0.047); had immunization information 98% (OR_MH_ 0.02, 95% CI 0.00–0.12, p = 0.001); with no obstacles accessing immunization services 98% (OR_MH_ 0.02, 95% CI 0.00–0.12, p < 0.001) and had good knowledge about immunization schedules 70% (OR_MH_ 0.30, 95% CI 0.15–0.61, p < 0.001) were more likely to comply with childhood vaccination. Knowledge of (EPI) services, including *RTS, S/AS01* malaria vaccine, was associated with high compliance to overall immunization services and schedules 78% (AOR 0.22, 95% CI 0.06–0.78, p = 0.019).

**Conclusion:**

Non-compliance with recommended EPI vaccinations, including *RTS,S/AS01*, was largely due to socio-economic factors, having information and knowledge about vaccines, immunization schedules, and access to immunization services. Healthcare providers can improve immunization coverage by emphasizing education on vaccines and vaccine schedules, using technologies and making services accessible in Ghana.

## Background

Immunization is crucial for safeguarding individuals against specific diseases, especially in developing countries where over 10 million children succumb to preventable illnesses annually [1]. The Expanded Programme on Immunization (EPI) serves as a pivotal link between children and healthcare systems, introducing vital interventions such as vaccines, Long-Lasting Insecticidal Nets (LLINs), de-worming, Vitamin A, and growth assessments [2]. Recognizing the significance of a healthy population in fostering economic growth, global efforts align with the United Nations 2030 Sustainable Development Goals to ensure universal access to vaccines for well-being at all ages. Childhood immunization programmes emerge as cost-effective interventions, diminishing infectious diseases and child mortality [3–5].

Timely vaccination is pivotal for saving lives and ensuring healthy futures for children [6, 7]. Despite high vaccination coverage, studies indicate that timeliness and acquired immunity can be deficient [4, 8].

Ghana's EPI, initiated in 1978, has significantly reduced infant mortality and morbidity rates and achieved elimination certification for maternal and neonatal tetanus in 2011 [9, 10]. The Volta Region, however, lags in health indicators, particularly immunization coverage of 87 per cent [11] Ghana's evolving EPI covers 13 Vaccine Preventable Diseases (VPDs), including the new *RTS,S/AS01* malaria vaccine [11–14].

The National EPI policy mandates specific vaccinations for children and women, adapting to changes such as the introduction of the pentavalent vaccine in 2002 [7, 11, 15]. Despite successes, challenges persist, with hard-to-reach areas facing barriers like the healthcare system and parental time constraints [16]. Full compliance with the EPI schedule is crucial for achieving vaccination goals and accurately estimating coverage for effective disease prevention in sub-Saharan Africa, including Ghana.

Global immunization coverage declined to 83% in 2020, attributed to the COVID-19 pandemic, leaving 23 million children unvaccinated, the highest since 2009 [17, 18]. Ghana Health Service (GHS) remains committed to universal immunization coverage, considering it a key health performance indicator. The EPI Program aims to reduce child morbidity, achieve high antigen coverage, and sustain disease elimination goals. Introducing the *RTS,S/AS01* malaria vaccine into the routine system initially faced challenges, including lower reporting of Adverse Events Following Immunization (AEFI) and concerns about severe side effects. Public awareness and trust-building are crucial to combat vaccine hesitancy. Caregiver knowledge of vaccines and caregivers'compliance with childhood immunization schedule, among others, have been noted as key factors in the acceptance of vaccination programmes. Other important factors include access to health services, distribution of health workers, time spent, and loss of earnings by caregivers when accessing vaccination services [6, 7, 10, 12, 14].

It is important to assess whether additional efforts should be made to improve caregiver knowledge and compliance with childhood immunization schedules. Interventions based on socio-cultural-economic and behavioural change of community members have been noted as key to the success of any programme. The effect of noncompliance with immunization schedules was a possible contributor to high dropout rates in immunization [19].

This study's overall aim was to assess caregivers'compliance with EPI immunization schedules, including the RTS,S/ASO1 malaria vaccine, in the Central Tongu district of Ghana to improve vaccination coverage.

## Methods

### Study design

A case–control study was conducted among caregivers of children aged 24–40 months who were not fully immunized (cases) and children of the same age group who were fully immunized as controls in the Central Tongu District of the Volta region of Ghana. The study area is one of the six regions piloting the RTS,S/AS01E malaria vaccine implementation in Ghana. Interviews with caregivers of case and control children were conducted in December 2021, approximately 34 months after the introduction of the *RTS,S/AS01E* malaria vaccine in Ghana.

### Study site description

The study area covers 700 square kilometres with an estimated population of 87,360 according to the 2021 Ghana Population and Housing Census. Subsistence farming is the main occupation of the population. Pottery and earthenware dishes are also made for the local market and exported to the district [20]. The health facilities in the study area, which provide curative and preventive services, include a hospital (1), health centres (5), private clinics (2), community-based health planning and services centres (14), and outreach points (176). EPI services are part of reproductive and child health services at all health facilities, mainly by Community Health Nurses (CHNs) and Community Health Officers (CHOs). Immunization coverage in the district was low during the COVID-19 pandemic, but coverage levels have increased to pre-pandemic levels. In 2022, about 75.4% of children in the district received 3 doses of diphtheria-tetanus-pertussis *Haemophilus influenzae* type b- hepatitis B (DPT-Hib-HeB3) vaccine, 74.4% of children received the first dose of measles vaccine, and 74.3% received yellow fever vaccine [17, 20].

### Selection of cases and controls

*Cases*: A list of children who assessed immunization services from November 2018 to March 2022 was obtained from the immunization register at the health facilities within the district. From this list, all those who did not complete their immunization schedule were compiled to serve as the case list, and those who completed their immunization schedule served as the control list. A systematic sampling technique was used to obtain a representative study sample. A list of all children 24–40 months who assessed EPI service within the period May 2019-March 2022 in the health facility register (sample frame). The total number of children obtained was divided by the sample size to get the K sampling interval. The first number on the list was randomly selected to start, followed by the Kth number until the sample size was attained. For example, in this study, a total of 116 cases were required. Suppose the total number of children 24–40 months who did not complete immunization in health facilities registers is 550, and the required sample size is 116. In that case, 550/116 gives a sampling interval estimated to be 5. The starting interval was from the 1 st to the 5th. If the starting point was chosen to be the 4th, the next case was the 9th, followed by the 14th, 19th, and 24th until a total sample of 116 cases was selected.

*Controls*: From the list of all children 24–40 months who completed their immunization schedule, each case was matched with a control by age, sex, and residence. Where there was more than one control with the same age, sex and place of residence, balloting was done to select one. The names or numbers were written on paper wrapped into balls, placed in a container, and mixed. A neutral person not involved in the study was asked to pick one wrapped paper. The name or the number selected was used to match that particular case.

### Sample size determination

This was determined through the open-source calculator—SSCC for case control studies [21]

The sample size was calculated using the following assumptions:

$$ {\text{N}}_{{{\text{Kelsey}}}}  = \left( {{\text{z}}_{{{\upalpha  \mathord{\left/ {\vphantom {\alpha  2}} \right. \kern-\nulldelimiterspace} 2}}}  + {\text{z}}_{\upbeta } } \right)^{2} {{{\text{p}}\left( {1 - {\text{p}}} \right)\left( {{\text{r + 1}}} \right)} \mathord{\left/ {\vphantom {{{\text{p}}\left( {1 - {\text{p}}} \right)\left( {{\text{r + 1}}} \right)} {\text{r}}}} \right. \kern-\nulldelimiterspace} {\text{r}}}\left( {{\text{p}}_{0}  - {\text{p}}_{1} } \right)^{2}  $$

1. The sample size, calculated with a two-sided confidence level (1-alpha) = 95% confidence level.
2. A power of (% chance of detecting) = 80%.
3. A ratio of controls to cases as 1.
4. Hypothetical proportion of controls with exposure = 97 (estimated national immunization coverage in children under five) [4].
5. Hypothetical proportion of cases with exposure: 86.61
6. A least extreme Odds Ratio to be detected of 0.2
7. yielded 110 controls and 110 cases giving a total of 220 [21]
8. Adding 5% non-response rate of 220 becomes 11 and so adding up the product of 11 to 220 yielding the total sample size 231 (cases = 116, controls = 116).

Therefore, a minimum sample size of 232 was required for the study.

### Data collection

A total of 116 children aged 24 to 40 months from health facilities registers in Central Tongu district who did not complete their immunization schedules between November 2018 to December 2021 were systematically selected as cases and 116 children who completed their immunization schedules from the registers within the same period matched on age, sex, and place of residence were selected as controls.

All vaccinated children are recorded in health facilities registers to monitor vaccination coverage and uptake in the district. Trained data collectors visited all caregivers of selected cases and control children aged 24 to 40 months from the health facility registers at home. During a home visit, the caregivers were asked by data collectors for detailed information on caregivers and their children, including socio-demographic characteristics and socio-economic information, caregiver knowledge of immunization schedule, attitude and acceptability of immunization against malaria, distance from health facility, attitude of health workers, availability of vaccines and health education. For children; date of birth, birth order, site of birth, sex, vaccination status, availability of home-based record of vaccination and any side effect following immunization. A photograph of the home-based record was taken. The data collectors used standardized data collection instruments and immunization posters or visual aids to ask about the caregivers'recall of the vaccines the child received.

### Key study definitions

*Cases*: Caregivers of children aged 24–40 months who accessed immunization services from November 2018 to March 2022 and did not receive the recommended WHO/EPI vaccine doses, considered to protect the child at an age-appropriate time from vaccine-preventable diseases.

*Controls*: Caregivers of children aged 24–40 months who accessed immunization services from November 2018 to March 2022 and had completed or are fully immunized based on the WHO/EPI recommended schedule.

*Complied to immunization schedule*: A child aged 24–40 months who received all recommended doses of all vaccines on the expected date or age-appropriate (a dose of BCG at birth, a dose of IPV at 14 weeks, four doses of OPV at birth, 6, 10, 14 weeks, three doses each of Pentavalent and PCV-13 at 6,10,14 weeks, two doses of Rotavirus vaccine at 6 and 10 weeks, one dose each of measles and yellow fever vaccines at 9 months, one dose of measles rubella at 18 months, one dose of meningococcal A at 18 months and 4 doses of *RTSS/AS01* malaria vaccine at 6, 7, 9, and 24 months).

*Non-compliant*: A combination of both partially immunized and non-vaccinated, with low uptake of the vaccine.

*Partially complied/immunized*: A child who did not receive one or more of the prescribed vaccine doses considered to offer protection at an age-appropriate for vaccine-preventable diseases**.**

*Fully immunized*: A child who received all doses of all vaccines on an expected date or age-appropriate. (a total of 12 vaccines and 23 doses at age 24 months).

### Data source

Data on immunization were collected from households using child health record cards. In cases where these cards were unavailable or vaccination was not recorded in the card, the caregiver’s immunization recall was accepted. In instances of immunization failure, the reasons were gathered from caregivers. For BCG, the presence or absence of a visible scar was also used as a source of information. An immunization poster or visual aid guided and elicited information on caregivers'recall of a child’s immunization status.

### Data management and analysis

The data was collected quantitatively, and the Principal Investigator (PI) ensured that questions were asked correctly and ethical and operational procedures were followed properly to ensure that information needed from the study participants was assessed correctly. Each questionnaire was assigned a unique code before data collection. At the end of the fieldwork, a secure database was created to contain all the information about the study participants. The data is only accessible to the PI and the supervisors. The data that has already been entered is protected with a password. The special codes assigned to the questionnaires were used for the data entry. This ensured confidentiality since participants’ names were not captured in the database. The PI and the supervisors were the only ones accessing the entered data. This is to ensure that the data is secured and protected.

### Data analysis

Data from the RedCap was cleaned and exported to Stata version 15.0 for analysis. Simple frequencies and percentages were used for categorical variables, and mean (SD) or median (IQR) for continuous variables. A Mantel–Haenszel odds ratio was used to assess the strength of the association between the level of compliance and other independent variables. Using a manual stepwise forward selection, significant variables were put in the multiple conditional logistic models, one at a time, to check for significant association until a final model of significant variables was achieved. Statistical significance was considered based on the p-value < 0.05 at a 95 percent confidence level, and results were displayed in tables and graphs.

## Results

A total of 220 caregivers of children aged 24–40 months were recruited for the study. One hundred and ten (50%) were cases, and 110 (50%) were controls. The mean age of caregivers was 30.38 ± 5.3 years. On ethnicity, the majority (92.7%, n = 204) of the caregivers were Ewes with Christian religion (95%, n = 209) and rural dwellers (97.3%, n = 214). A higher proportion of the caregivers had formal education (93.2%, n = 205), were employed (95.0%, n = 209) and earned income (75.9%, n = 167). About half of caregivers were married (52.3%, n = 115). The majority of the caregivers had gravidity of one or more (98.6%, n = 217), with parity between 0 and 2 and family size of ≤ 5 people (62.7%, n = 138) (Table 1). Thirty-nine percent (n = 70) of the caregivers had poor information about immunization (65 cases and 5 controls).

**Table 1:** Background characteristics of caregivers of children in Central Tongu of Ghana

Variable	Case (110) n (%)	Control (110) n (%)	Total (220) N (%)	χ^2^ (p-value)
Mean age (S.D)	30.52 (5.0)	30.24 (5.6)	30.38 (5.3)	
Age Group				
≤ 30	60 (54.6)	65 (59.1)	125 (56.8)	
31 +	50 (45.4)	45 (40.9)	95 (43.2)	0.67 (0.414)
Educational level				
No formal/read and write	10 (9.1)	5 (4.6)	15 (6.8)	
Educated	100 (90.9)	105 (95.4)	205 (93.2)	1.79 (0.181)
Occupation				
Unemployed	7 (6.4)	4 (3.6)	11 (5.0)	
Employed	103 (93.6)	106 (96.4)	209 (95.0)	0.86 (0.353)
Marital Status				
Not married	51 (46.4)	54 (49.1)	105 (47.7)	
Married	59 (53.6)	56 (50.9)	115 (52.3)	0.16 (0.686)
Income				
No/unknown/undisclosed	30 (27.3)	23 (20.9)	53 (24.1)	
Yes	80 (72.7)	87 (79.1)	167 (75.9)	1.22 (0.270)
Ethnicity				
Akan/Hausa	9 (8.2)	7 (6.4)	16 (7.3)	
Ewe	101 (91.8)	103 (93.6)	204 (92.7)	0.27 (0.604)
Religion				
Muslim/Traditionalist	8 (7.3)	3 (2.7)	11 (5.0)	
Christian	102 (92.7)	107 (97.3)	209 (95.0)	(0.215)*
Place of Residence				
Urban	4 (3.6)	2 (1.8)	6 (2.7)	
Rural	106 (96.4)	108 (98.2)	214 (97.3)	0.68 (0.408)
Gravidity				
None	0 (0.0)	3 (2.7)	3 (1.4)	
1 +	110 (100.0)	107 (97.3)	217 (98.6)	3.04 (0.081)
Parity				
0–2	66 (60.0)	74 (67.3)	140 (63.6)	
3 +	44 (40.0)	36 (32.7)	80 (36.4)	1.26 (0.262)
Family size				
≤ 5	65 (59.1)	73 (66.4)	138 (62.7)	
> 5	45 (40.9)	37 (33.6)	82 (37.3)	1.24 (0.265)

^*^Fisher’s Exact

The main factors that contributed to poor information among the cases included unawareness of the need to return for subsequent doses, unawareness of the need for immunization, fear of side reactions, unawareness of the next clinic session, unawareness of session rescheduled dates and wrong ideas about contraindications (Table 2). Overall, 31% (n = 68) of the caregivers experienced obstacles while accessing immunization services. Of these, 94% were cases (n = 64). The main contributing obstacles encountered were the mother being too busy 19.1% (n = 42), family problems 7.7% (n = 17), inconveniences with the immunization schedule 4.5% (n = 10) and child illness during immunization day 2.7% (n = 6) (Table 3).

**Table 2:** Assessment of information on vaccines and vaccination schedules among caregivers

Variable	Case (110) n (%)	Control (110) n (%)	Total (220) N (%)	χ^2^ (p-value)
Unaware of need for immunization				
No	94 (85.4)	110 (100.0)	204 (92.7)	
Yes	16 (14.6)	0 (0.0)	16 (7.3)	(< 0.001)*
Unaware of need to return for consequent doses				
No	94 (85.4)	108 (98.2)	202 (91.8)	
Yes	16 (14.6)	2 (1.8)	18 (8.2)	(0.001)*
Place and time of immunization unknown				
No	105 (95.4)	110 (100.0)	215 (97.7)	
Yes	5 (4.6)	0 (0.0)	5 (2.3)	(0.060)*
Fear of side reactions				
No	95 (86.4)	110 (100.0)	205 (93.2)	
Yes	15 (13.6)	0 (0.0)	15 (6.8)	(< 0.001)*
Wrong ideas about contraindications				
No	99 (90.0)	110 (100.0)	209 (95.0)	
Yes	11 (10.0)	0 (0.0)	11 (5.0)	(0.001)*
Unaware of the next clinic session				
No	98 (89.1)	107 (97.3)	205 (93.2)	
Yes	12 (10.9)	3 (2.7)	15 (6.8)	(0.029)*
Unaware of session rescheduled				
No	100 (90.9)	108 (98.2)	208 (94.5)	
Yes	10 (9.1)	2 (1.8)	12 (5.5)	(0.034)*
Unexpectedly and not told verbally on next clinic session				
No	101 (91.8)	109 (99.1)	210 (95.4)	
Yes	9 (8.2)	1 (0.9)	10 (4.6)	(0.019)*
Overall Information				
Good information	45 (40.9)	105 (95.4)	150 (68.2)	
Poor information	65 (59.1)	5 (4.6)	70 (31.8)	**75.43 (< 0.001)**

^*^Fisher’s Exact

**Table 3:** Assessment of obstacles confronting caregivers in accessing immunization services

Variable	Case (110) n (%)	Control (110) n (%)	Total (220) N (%)	χ^2^ (p-value)
Place of immunization too far				
No	108 (98.2)	110 (100.0)	218 (99.1)	
Yes	2 (1.8)	0 (0.0)	2 (0.9)	(0.498)*
Inconvenience in the immunization schedule				
No	100 (90.9)	110 (100.0)	210 (95.4)	
Yes	10 (9.1)	0 (0.0)	10 (4.5)	(0.002)*
Mother too busy				
No	70 (63.6)	108 (98.2)	178 (80.9)	
Yes	40 (36.4)	2 (1.8)	42 (19.1)	(< 0.001)*
Family problems				
No	93 (84.5)	110 (100.0)	203 (92.3)	
Yes	17 (15.5)	0 (0.0)	17 (7.7)	(< 0.001)*
Child ill—not brought				
No	109 (99.1)	110 (100.0)	219 (99.5)	
Yes	1 (0.9)	0 (0.0)	1 (0.5)	(1.000)*
Child ill—brought but not given immunization				
No	104 (94.5)	110 (100.0)	214 (97.3)	
Yes	6 (5.5)	0 (0.0)	6 (2.7)	(0.029)*
Long waiting time				
No	110 (100.0)	109 (99.1)	219 (99.5)	
Yes	0 (0.0)	1 (0.9)	1 (0.5)	(1.000)*
No means of transport				
No	103 (93.6)	109 (99.1)	212 (96.4)	
Yes	7 (6.4)	1 (0.9)	8 (3.6)	(0.065)*
Vaccination session not available at all times				
No	105 (95.4)	109 (99.1)	214 (97.3)	
Yes	5 (4.6)	1 (0.9)	6 (2.7)	(0.212)*
Overall obstacle in accessing immunization services				
No obstacle	46 (41.8)	106 (96.4)	152 (69.1)	
Obstacle	64 (58.2)	4 (3.6)	68 (30.9)	**(< 0.001)**

^*^Fisher’s Exact

There was good knowledge about vaccines and vaccination schedules, 62% (n = 139). This was noted more among control respondents (58.4% n = 80) compared to cases (41.6% n = 57). A little over half (58.2%, n = 128) of the respondents knew that vaccination protects against preventable diseases such as polio and measles and keeps children healthy (59%). The majority of respondents (75.0%, n = 165) knew vaccination starts as soon as the child is born and is completed within 24 months (60.0%, n = 132) (Table 4). Caregivers who earn income, had information about immunization schedules, with no obstacles in accessing immunization services and had good knowledge of immunization schedules were 46% [OR_MH_ = 0.54 (95% CI 0.29–1.00), p = 0.047], 98% [OR_MH_ = 0.02 (95% CI 0.00–0.12), p < 0.001], 98.0% [OR_MH_ = 0.02 (95% CI 0.00–0.12), p < 0.001] and 70% [OR_MH_ = 0.30 (95% CI 0.15–0.61), p < 0.001] less likely to be non-compliant to EPI and RTS,S/AS01 vaccination schedules (Table 5), respectively.

**Table 4:** MH Association between socio-demographic characteristics and non-compliance to EPI and RTS,S/AS01 vaccination schedule

Variables		Control		Total	OR_MH_	P-value	95% CI
Mother’s age group	Case	≤ 30	31 +				
	≤ 30	37	23	60	0.82	0.484	0.47–1.43
	31 +	28	22	50			
	Total	65	45	110			
Ethnicity	Case	Ewe	Non-Ewe				
	Ewe	94	7	101	0.78	0.617	0.29–2.09
	Not ewe	9	0	9			
	Total	103	7	110			
Religion	Case	Christian	Non-Christian				
	Christian	100	2	102	0.28	0.096	0.06–1.37
	Not Christian	7	1	8			
	Total	107	3	110			
Residence	Case	Rural	Urban				
	Rural	104	2	106	0.50	0.414	0.09–2.73
	Urban	4	0	4			
	Total	108	2	110			
Educational level	Case	Formal Education	No formal education				
	Formal Education	95	5	100	0.50	0.197	0.17–1.46
	No formal education	10	0	10			
	Total	105	5	110			
Earn Income	Case	Yes	No				
	Yes	62	15	77	0.54	**0.047**	**0.29–1.00**
	No	28	5	33			
	Total	90	20	110			
Occupation	Case	Employed	Not employed				
	Employed	99	4	103	0.57	0.366	0.17–1.95
	Not employed	7	0	7			
	Total	106	4	110			
Marital status	Case	Married	Not married				
	Married	31	28	59	1.12	0.680	0.65–1.92
	Not married	25	26	51			
	Total	56	54	110			
Gravidity	Case	1 +	None				
	1 +	107	3	110	7.00	0.134	0.36–135.50
	None	0	0	0			–
	Total	107	3	110			–
Parity	Case	0–2	3 +				–
	0–2	49	17	66			–
	3 +	25	19	44			–
	Total	74	36	110			–
Family size	Case	≤ 5	> 5				
	≤ 5	43	23	66	0.77	0.336	0.44–1.32
	> 5	30	14	44			–
	Total	73	37	110			–
Other variables							
Information about vaccination	Case	Informed	Not informed				
	Informed	44	1	45	**0.02**	** < 0.001**	**0.00–0.12**
	Not informed	61	4	65			–
	Total	105	5	110			–
Obstacle to vaccination	Case	No obstacle	Obstacle				
	No obstacle	45	1	46	**0.02**	** < 0.001**	**0.00–0.12**
	Obstacle	61	3	64			–
	Total	106	4	110			–
Knowledge about vaccination	Case	Good recall	Poor recall				–
	Good knowledge	47	10	57	**0.30**	** < 0.001**	**0.15–0.61**
	Poor knowledge	33	20	53			–
	Total	80	30	110

**Table 5:** Conditional logistic regression of factors associated with EPI and RTS,S/AS01 vaccination and the odds of non-compliance to vaccine schedule

Variable	Control (N = 110) n (%)	Case (N = 110) n (%)	χ^2^ (p-value)/Fisher’s Exact	COR [95% CI], P-value	AOR [95% CI], P-value
Age group					
≤ 30	66 (60.0)	60 (54.5)		Ref	Ref
31 +	44 (40.0)	50 (45.5)	0.67 (0.414)	1.27 (0.72–2.22), 0.397	–
Level of education					
No formal education	5 (4.6)	10 (9.1)		Ref	Ref
Formal education	105 (95.4)	100 (90.9)	1.79 (0.181)	0.50 (0.17–1.46), 0.206	–
Occupation					
Unemployed	4 (3.6)	7 (6.4)		Ref	Ref
Employed	106 (96.4)	103 (93.6)	(0.538)	0.57 (0.17–1.95), 0.372	–
Ethnicity					
Akan/Hausa	7 (6.4)	9 (8.2)		Ref	Ref
Ewe	103 (93.6)	101 (91.8)	0.27 (0.604)	0.78 (0.29–2.09), 0.618	–
Religion					
Islam/Traditionalist	3 (2.7)	8 (7.3)		Ref	Ref
Christian	107 (97.3)	102 (92.7)	(0.215)	0.28 (0.06–1.37), 0.118	–
Residence					
Urban	2 (1.8)	4 (3.6)		Ref	Ref
Rural	108 (98.2)	106 (96.4)	(0.683)	0.50 (0.09–2.73), 0.423	–
Income					
No/unknown/undisclosed	23 (20.9)	30 (27.3)		Ref	Ref
Yes	87 (79.1)	80 (72.7)	1.22 (0.270)	0.72 (0.39–1.32), 0.288	–
Marital status					
Not married	54 (49.1)	51 (46.4)		Ref	Ref
Married	56 (50.9)	59 (53.6)	0.16 (0.686)	1.12 (0.65–1.92), 0.680	–
Parity					
0–2	74 (67.3)	66 (60.0)		Ref	Ref
3 +	36 (32.7)	44 (40.0)	1.26 (0.262)	1.47 (0.79–2.72), 0.220	–
Family size					
≤ 5	73 (66.4)	65 (59.1)		Ref	Ref
> 5	37 (33.6)	45 (40.9)	1.24 (0.265)	1.36 (0.79–2.36), 0.269	–
Information					
Informed	105 (95.4)	45 (40.9)		Ref	Ref
Uninformed	5 (4.6)	65 (59.1)	**75.43 (< 0.001)**	**61.00 (8.46–440.03), < 0.001**	8.31 (0.08–820.09), 0.366
Obstacle					
No obstacle	106 (96.4)	46 (41.8)		Ref	Ref
Obstacle	4 (3.6)	64 (58.2)	**76.63 (< 0.001)**	**61.00 (8.46–440.03), < 0.001**	8.31 (0.08–820.09), 0.366
Overall knowledge					
Poor knowledge	30 (27.3)	53 (48.2)		Ref	Ref
Good knowledge	80 (72.7)	57 (51.8)	**10.23 (0.001)**	**0.30 (0.15–0.61), 0.001**	**0.29 (0.10–0.80), 0.018**

## Discussion

In this study in Ghana, knowledge about immunization services and vaccination schedules was good (70%). Socio-economic and health systems factors affected compliance with immunization services and schedules. Compliance with EPI services, including receipt of RTSS malaria vaccines, was high based on the availability of information on immunization services, easy access to health services and knowledge about vaccination schedules. It was also found that caregivers with general knowledge of EPI services, including RTS,S/AS01 malaria vaccine schedules, were more likely to comply with immunization services and schedules.

The relationship between socioeconomic and health service-related factors and compliance with immunization services has been reported in low and middle-income countries such as Nigeria, Kenya and Ethiopia, where studies found that mothers with higher education levels were more likely to complete their children's immunization series [1, 22–25]. A study in Kenya found that distance to health facilities was a significant barrier to immunization access [4, 26] and Ethiopia found that children living in rural areas were less likely to receive immunizations compared to those in urban areas [27]. However, this study is the first to report data from the Volta region of Ghana after the implementation of the malaria vaccine programme in Ghana in 2019. In this study, poor caregivers were more likely to comply with EPI services, including vaccine schedules, than income earners. Reports of caregivers who earned a salary had more influence in decision-making, and therefore, have improved child immunization status, have been published [28]. Two studies from Ethiopia and Ghana have reported the relationship between socioeconomic status and children fully vaccinated with the recommended basic EPI vaccines. In another study, caregivers who earned income were more likely to have their children fully vaccinated compared to caregivers who did not earn any income [29].

This study found that caregivers with information about immunization schedules were less likely to have failed to complete the EPI and *RTS,S/AS01* immunization schedule. A study in Saudi Arabia reported that caregivers seek information regarding their children’s vaccinations from healthcare workers and their MOH call centre [30]. Another report from the Jordanian Ministry of Health [1] indicated a system in the public health centres in the country where each registered child is given a written vaccination record (vaccination card) containing information about the dates of vaccinations received and upcoming vaccines. Each of the children also has an electronic medical file that includes health information, growth and development information, and immunization schedule, providing access to information on immunization.

In the present study, caregivers with no obstacles were less likely to have failed to complete the EPI and *RTS,S/AS01* immunization schedule. These findings are consistent with recent studies that reported distance to health facilities, mode of transportation, and accessibility to vaccination sites as obstacles to vaccination services [1, 3].

In this present study, caregivers who had good knowledge about EPI, including RTS,S/AS01 vaccine and immunization schedules, were less likely to have defaulted in receiving the required vaccination. This finding is consistent with a published study from Nigeria, which reported that caregiver knowledge of childhood immunization and schedules was a key determinant of compliance with immunization [31].

## Strengths and limitations

This study has limitations. In-depth qualitative interviews to explore the experiences of caregivers and health service providers could not be conducted. Vaccination status was recorded through caregiver recall of vaccines when vaccination cards were unavailable. However, the authors did not rely on recall alone; visual aids on EPI vaccines were used to clarify the response. Caregivers were also blinded to the study questions about determinants of compliance with vaccination, and all questions on determinants were asked before questions on vaccines. The conduct of the study in one district in Ghana reduces the generalizability of our study a little, but is unlikely to have introduced any bias. The strength of the study was that recall bias could be controlled by using the immunization poster to guide the recall process.

## Conclusion

This study appears to be the first to analyse the district-level influence of socioeconomic and health system factors on compliance with EPI vaccines, including the RTS,S/AS01 malaria vaccine, after the introduction of the *RTS,S/AS01* malaria vaccine in Ghana. This study indicates that caregivers with good knowledge about EPI, including RTS,S/AS01 malaria vaccine, and immunization schedules, had good information and no obstacles, were less likely not to comply to receive the required vaccination. The government of Ghana, policy makers, non-governmental organization, health directorates, and all health workers (Health promotion officers, Public Health Nurses, Community Health nurses, Community Health Volunteers) should intensify health education through all available channels especially as radio, community radio or information centre and home visit in the district. Collaborates with agencies to utilize technology to improve caregiver’s knowledge and compliance on immunization services. Provision of resources to health promotion team to lead health education activities on immunization in the district, to increase compliance to age-appropriate for EPI & RTS,S/AS01 schedule that is needed to prevent future outbreak of diseases, provide optimum protection & herd immunity against vaccine-preventable diseases.

## Data Availability

No datasets were generated or analysed during the current study.

## Abbreviations

BCG: Bacillus Calmette et Guerin
CEM Ghana: Consortium to Evaluate Mosquirix in Ghana
CHNs: Community Health Nurses
CHOs: Community Health Officers
CT-DHD: Central Tongu District Health Directorate
EPI: Expanded programme on immunization
FNBSPH: Fred Newton Binka School of Public Health, University of Health and Allied Sciences
IQR: Inter Quartile Range
KHRC: Kintampo Health Research Centre
LLINs: Long-Lasting Insecticidal Nets
MVPETS: Malaria Vaccine Pilot Evaluation Training Scholarship
PI: Principal Investigator
SD: Standard Deviation or median
UHAS-REC: University of Health and Allied Sciences (UHAS) Research Ethics Committee
WHO: World Health Organization
